# Structural, Thermal, and Optical Properties of Er^3+^-Doped Lead Tungstates and Lead Molybdate–Tungstates

**DOI:** 10.3390/ma19214661

**Published:** 2026-11-02

**Authors:** Elżbieta Tomaszewicz, Magdalena Kotfica, Hubert Fuks, Paweł Kochmański

**Affiliations:** 1 Department of Inorganic and Analytical Chemistry, Faculty of Chemical Technology and Engineering, West Pomeranian University of Technology in Szczecin, Al. Piastów 42, 71-065 Szczecin, Poland; magdalena.kotfica@zut.edu.pl; 2 Faculty of Mechanical Engineering and Mechatronics, West Pomeranian University of Technology in Szczecin, Al. Piastów 19, 70-310 Szczecin, Poland; hubert.fuks@zut.edu.pl (H.F.); pawel.kochmanski@zut.edu.pl (P.K.)

**Keywords:** Er^3+^ ion, scheelites, solid solution, thermal stability, optical properties

## Abstract

New Er^3+^-doped lead tungstates and molybdate–tungstates with the chemical formulae Pb_1−3*x*_▯*_x_*Er_2*x*_WO_4_ (0 < *x* ≤ 0.0283) and Pb_1-3*x*_▯_x_Er_2*x*_(MoO_4_)_1-3*x*_(WO_4_)_3*x*_ (0 < *x* ≤ 0.0536), where ▯ denotes a vacant crystallographic site, were successfully synthesized via a high-temperature solid-state reaction method using PbWO_4_ or PbMoO_4_ and Er_2_(WO_4_)_3_ as starting reactants. X-ray diffraction and scanning electron microscopy analyses confirmed the formation of single-phase, tetragonal scheelite-type materials (space group *I*4_1_/*a*) with an average crystallite size ranging from approximately 50 to 100 μm. The Er^3+^-doped ceramic materials remained stable up to their melting points and did not undergo any polymorphic transformation. Their melting points were lower than those of the corresponding scheelite-type matrix, namely, PbWO_4_ and PbMoO_4_. UV–Vis–NIR diffuse reflectance measurements demonstrated that all Er^3+^-doped materials are insulating and exhibit indirect optical transitions. The incorporation of Er^3+^ leads to a reduction in the optical band gap energy, from 3.73 to 3.47 eV for the doped tungstates and from 3.17 to 3.01 eV for the doped molybdate–tungstates, accompanied by a simultaneous increase in the Urbach energy. The refractive indices estimated using seven different empirical models exhibit only a weak dependence on Er^3+^ concentration and remain relatively high (approximately 2.15–2.50) for both material series.

## 1. Introduction

Doped lead tungstate (PbWO_4_) and lead molybdate (PbMoO_4_) are important inorganic materials that have attracted considerable attention due to their excellent luminescent, optical, and scintillation properties. The incorporation of transition-metal or rare-earth ions into these host lattices significantly modifies their structural and spectroscopic characteristics, enabling their application in various advanced technologies.

Doped lead tungstate is widely recognized as a high-performance scintillation material. Due to its high density, short radiation length, and fast scintillation response, PbWO_4_ crystals are extensively used in high-energy physics experiments for the detection of high-energy particles and γ-rays. Rare-earth doping enhances the efficiency of scintillation, light yield, and radiation hardness, improving detector performance in demanding environments [1,2,3,4,5,6,7,8].

Lead tungstate and lead molybdate doped with RE^3+^ ions are also employed as phosphor materials in light-emitting devices. Their strong luminescence under ultraviolet excitation makes them suitable for white light-emitting diodes (WLEDs), display technologies, and fluorescent lighting. By selecting appropriate dopant ions and their concentration, the emission color and intensity can be precisely controlled [9,10,11,12,13,14].

One of the most important applications of doped PbWO_4_ and PbMoO_4_ is in solid-state laser systems. Rare-earth ions such as Nd^3+^ or Yb^3+^ act as luminescent centers, producing efficient emission in the visible and near-infrared spectral regions. These materials are considered promising candidates for laser hosts, optical amplifiers, and wavelength conversion devices [15,16,17]. Another important area of application is optical sensing and photonic devices. The excellent transparency and nonlinear optical properties of these crystals, combined with the characteristic emission of rare-earth ions, make them attractive for optical sensors, optical temperature measurements, and frequency conversion devices. The high-temperature stability and relatively high refractive index of these materials further enhance their performance in integrated photonic systems [14,16,18].

Lead tungstate and lead molybdate are inorganic compounds that crystallize in the scheelite-type tetragonal structure with the space group *I*4_1_/*a* (No. 88) under ambient conditions [19,20,21]. In the scheelite-type structure, each W^6+^ ion in PbWO_4_, or Mo^6+^ ion in PbMoO_4_, is tetrahedrally coordinated by four oxygen ions, forming isolated WO_4_^2−^ or MoO_4_^2−^ tetrahedra. These polyhedra are linked through the surrounding lead ions rather than by direct sharing of oxygen ions, resulting in a three-dimensional framework [19,20,21]. The divalent lead ions occupy distorted eightfold-coordinated sites and are surrounded by eight oxygen ions, forming irregular PbO_8_ polyhedra [19,20,21].

Previous studies have demonstrated that Er^3+^ can substitute for Pb^2+^ in the PbWO_4_ and PbMoO_4_ lattices [22,23,24,25,26,27,28]. To date, most investigations have focused on Er-containing PbWO_4_ or PbMoO_4_ single crystals [22,23,24,25,26,27], whereas comparatively limited attention has been devoted to Er^3+^-doped nanocrystalline materials [28].

Huang et al. [22,23,24] investigated in PbWO_4_:Er^3+^ single crystals energy transfer from the host lattice to Er^3+^ ions, together with the effects of crystal orientation, dopant concentration, and thermal treatment on the emission characteristics. In particular, emission around 1.53 μm, corresponding to the ^4^I_13/2_ → ^4^I_15/2_ transition of Er^3+^, has been reported, as well as visible up-conversion bands centered at approximately 528, 550, and 660 nm under infrared excitation [22,23,24].

For PbMoO_4_:Er^3+^ single crystals, spectroscopic transition intensities and radiative parameters have been analysed using the Judd–Ofelt theory [25,26,27]. Subsequent studies have demonstrated room-temperature NIR emission near 1550 nm and green up-conversion emission around 520 and 550 nm under excitation at approximately 980 nm, with excited-state absorption proposed as a contributing up-conversion mechanism [25,26,27]. Furthermore, spatially resolved measurements have shown that the observed green up-conversion emission depends on the relative positions of the excitation and detection regions, highlighting the importance of measurement geometry when interpreting the luminescence response [25,26,27].

Hosseinpour et al. [28] prepared PbWO_4_:Er^3+^ nanoparticles using a co-precipitation procedure, and the effect of the calcination atmosphere (air and argon) on the luminescence properties and scintillation responses was investigated.

The main novelty of this work lies in confirming the formation, via a solid-state reaction method, of substitutional and vacancy solid solutions, as well as in establishing the models of these solutions (Pb_1-3*x*_▯*_x_*Er_2*x*_WO_4_ and Pb_1-3*x*_▯_x_Er_2*x*_(MoO_4_)_1-3*x*_(WO_4_)_3*x*_) and their homogeneity ranges. In contrast to previous studies focused primarily on the synthesis and luminescent characterization of Er^3+^-doped PbWO_4_ nanoparticles, the present approach considers Er^3+^ substitution together with the formation of cation vacancies (▯) required for charge compensation, providing a more comprehensive description of the defect chemistry of the investigated systems. Our studies cover a relatively broad range of Er^3+^ contents and enable a systematic assessment of the evolution of the crystal structure with increasing Er^3+^content. A key aspect of the present study is also the integration of structural, morphological, and optical data. The combined results of PXRD, FE-SEM, DTA-TG, and UV-Vis-NIR analyses enable the identification of relationships between Er^3+^ incorporation, cation-vacancy formation, crystal lattice distortions, and thermal and optical properties. Such a comprehensive structure–property analysis provides deeper insight into the role of Er^3+^ ions in modifying the properties of PbWO_4_ and PbWO_4_(MoO_4_)-based solid solutions.

## 2. Materials and Methods

### 2.1. Synthesis of PbErWO and PbErMoWO Solid Solutions

Microcrystalline samples of the new Pb_1−3*x*_▯*_x_*Er_2*x*_WO_4_ (*x* = 0.0098, 0.0283; hereafter referred to as PbErWO) and Pb_1−3*x*_▯*_x_*Er_2*x*_(MoO_4_)_1−3*x*_(WO_4_)_3*x*_ (*x* = 0.0098, 0.0283, 0.0455, and 0.0536; hereafter referred to as PbErMoWO) solid solutions, where ▯ means vacant sites, were synthesized via two-step synthesis. In both steps, a high-temperature solid-state reaction between appropriate reactants was applied. The following initial reactants were used in the first step of synthesis: lead oxide (PbO), molybdenum oxide (MoO_3_), tungsten oxide (WO_3_) (all raw materials of high-purity grade min. 99.95%, Alfa Aesar and without thermal pre-treatment), and erbium oxide (Er_2_O_3_, the purity 99.99% Alfa Aesar, heated at 900 °C during 12 h to remove traces of moisture and adsorbed CO_2_).

Lead tungstate (PbWO_4_), lead molybdate (PbMoO_4_), and erbium tungstate (Er_2_(WO_4_)_3_) were obtained analogously to our previous studies [29,30,31,32]. In the next step, two Er_2_(WO_4_)_3_/PbWO_4_ and four Er_2_(WO_4_)_3_/PbMoO_4_ mixtures with the content of erbium tungstate ranging from 1.00 to max. 6.00 mol% were prepared. The composition of all prepared mixtures is given in Table S1. The initial mixtures were sintered in corundum crucibles, in several 12 h heating stages, in air atmosphere, and at temperatures ranging from 800 to max. 990 °C. After each sintering period, the mixtures were slowly cooled down to ambient temperature, weighed, homogenized in a porcelain mortar, and examined using the powder X-ray diffraction (PXRD) method. A slight mass loss (not higher than 0.10%) was observed for each initial mixture during its heating. This observation clearly shows that the synthesis of new PbErWO and PbErMoWO solid solutions runs practically without mass change. This process can be described according to the following equations:

(1 − 3*x*) PbWO_4_ + *x* Er_2_(WO_4_)_3_ = Pb_1-3*x*_▯*_x_*Er_2*x*_WO_4_ (1)

(1 − 3*x*) PbMoO_4_ + *x* Er_2_(WO_4_)_3_ = Pb_1-3*x*_▯*_x_*Er_2*x*_(MoO_4_)_1-3*x*_(WO_4_)_3*x*_ (2)

**Figure 1:**
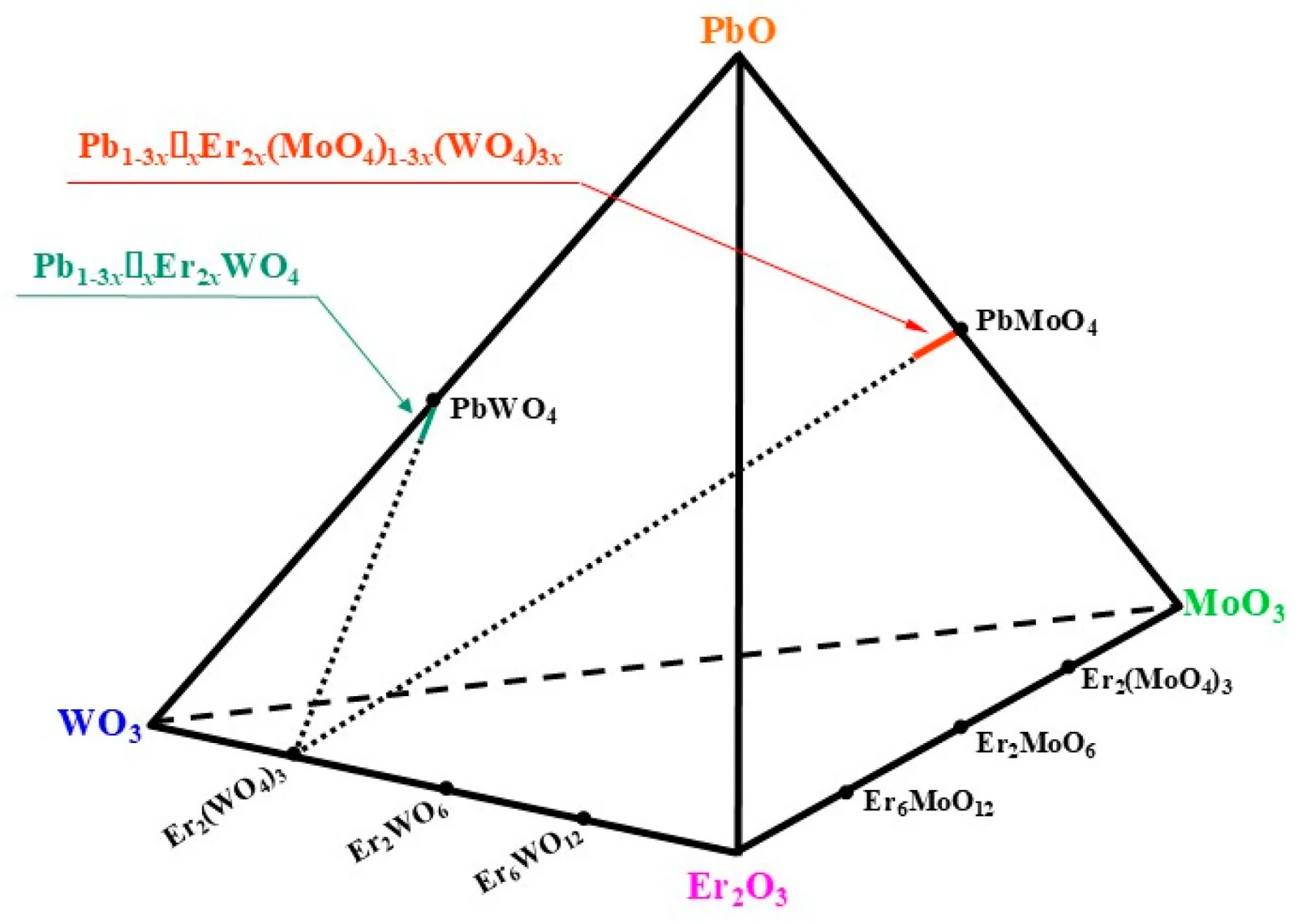
Tetrahedron of the PbO-WO_3_-Er_2_O_3_-MoO_3_ system with the location of the Pb_1-3*x*_▯*_x_*Er_2*x*_WO_4_ and Pb_1-3*x*_▯*_x_*Er_2*x*_(MoO_4_)_1-3*x*_(WO_4_)_3*x*_ solid solutions.

The formula of each obtained sample is shown in Table S1 and the locations of both solid solutions are marked in the tetrahedron of the PbO-WO_3_-Er_2_O_3_-MoO_3_ system (Figure 1).

### 2.2. Characterization of Methods

Powder X-ray diffraction (PXRD) measurements were conducted using an EMPYREAN II diffractometer (PANalytical, Almelo, The Netherlands) equipped with CuKα1,2 radiation (λ = 1.5418 Å). Diffraction patterns of all powdered samples were collected over the 2Theta range of 10–100° with a step size of 0.013°. The obtained data were processed using HighScore Plus 4.9a software, while the unit cell parameters were determined with the aid of POWDER 2.0 [33,34] and DICVOL 4.0 software [31].

The density of powder materials was measured using an Ultrapyc 1200e ultrapycnometer (Quantachrome Instruments, Boynton Beach, FL, USA) with nitrogen gas (purity 99.99%) as the pycnometric medium.

Thermal behavior of the samples was investigated via simultaneous differential thermal analysis (DTA), thermogravimetric analysis (TG), and differential scanning calorimetry (DSC) using an SDT 650 thermoanalyzer (TA Instruments, New Castle, DE, USA). The measurements were performed in air within the temperature range of 20–1200 °C at a constant heating rate of 10 K min^−1^. Corundum crucibles were employed throughout the experiments, and the mass of each analyzed specimen was approximately 30 mg.

The microstructure, particle morphology, and elemental composition of the PbErWO and PbErMoWO samples were characterized via field-emission scanning electron microscopy (FE-SEM) using a Hitachi SU-70 microscope (Hitachi, Naka, Japan) coupled with a NORAN™ System 7 energy-dispersive X-ray spectroscopy (EDS) system (Thermo Fisher Scientific, Madison, WI, USA) equipped with an UltraDry detector. SEM observations were carried out at an accelerating voltage of 10 kV using the secondary electron imaging mode. Prior to the measurements, the samples were coated with a thin palladium–gold layer deposited via the physical vapor deposition (PVD) thermal evaporation technique using a JEOL JEE-4X evaporator (JEOL, Tokyo, Japan) to ensure sufficient electrical conductivity.

Diffuse reflectance spectra in the UV–Vis–NIR region were collected with a JASCO V-670 spectrophotometer (JASCO International Co., Ltd., Hachioji, Tokyo, Japan) equipped with an integrating sphere. The radiation sources were a deuterium lamp for the UV range and a halogen lamp for the Vis-NIR range. Spectral measurements were performed over the wavelength range of 200–1000 nm using a scanning interval of 0.5 nm and a scan speed of 100 nm min^−1^.

## 3. Results and Discussion

### 3.1. X-Ray Diffraction Studies of PbErWO and PbErMoWO Materials

**Figure 2:**
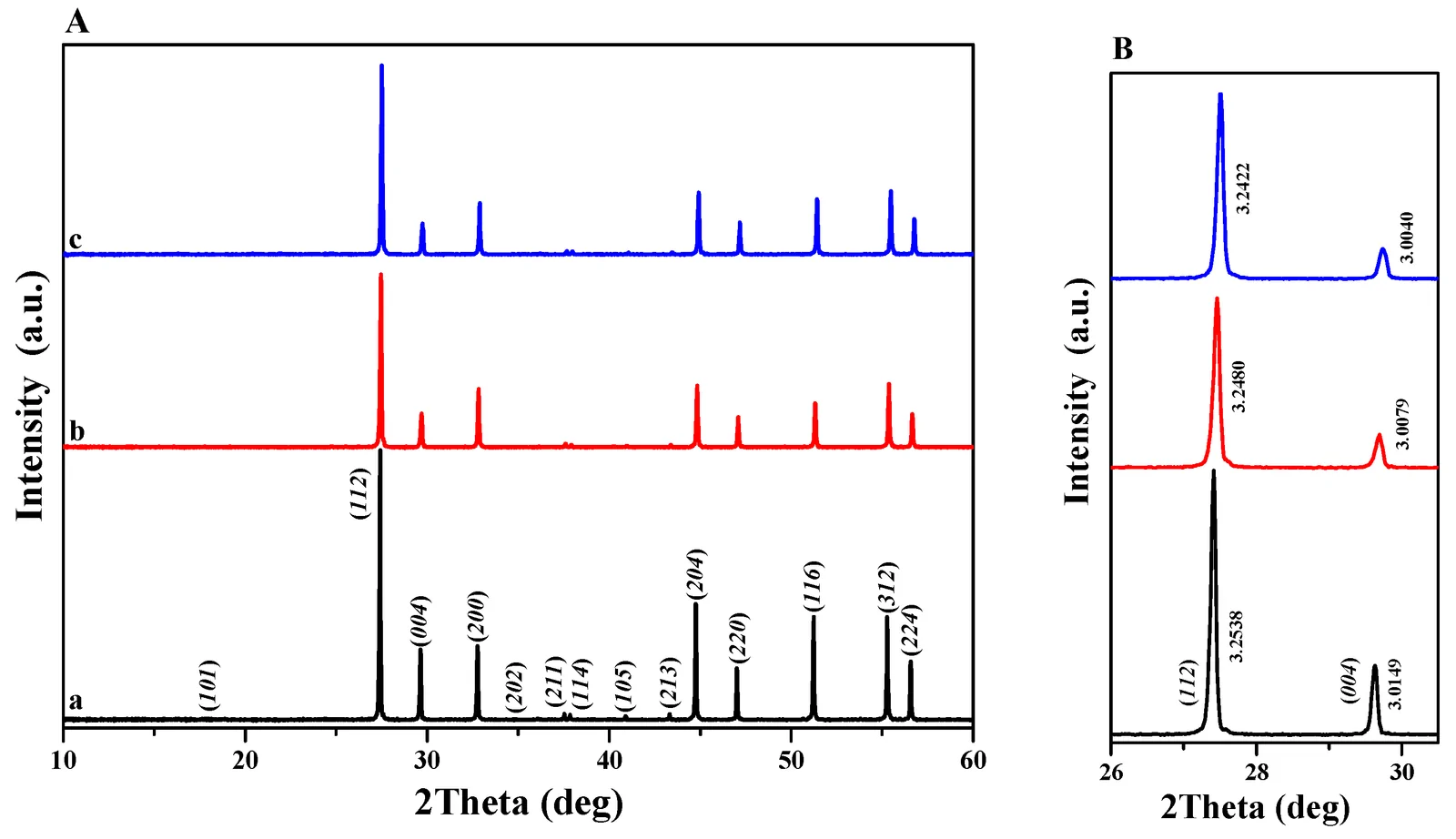
(**A**) Powder XRD patterns of PbWO_4_ (a) and PbErWO samples when *x* = 0.0098 (b) and *x* = 0.0283 (c); (**B**) powder diffraction patterns in the 2Theta range from 26 to 30.5° for (112) and (004) diffraction lines, as well as their corresponding interplanar distances.

**Figure 3:**
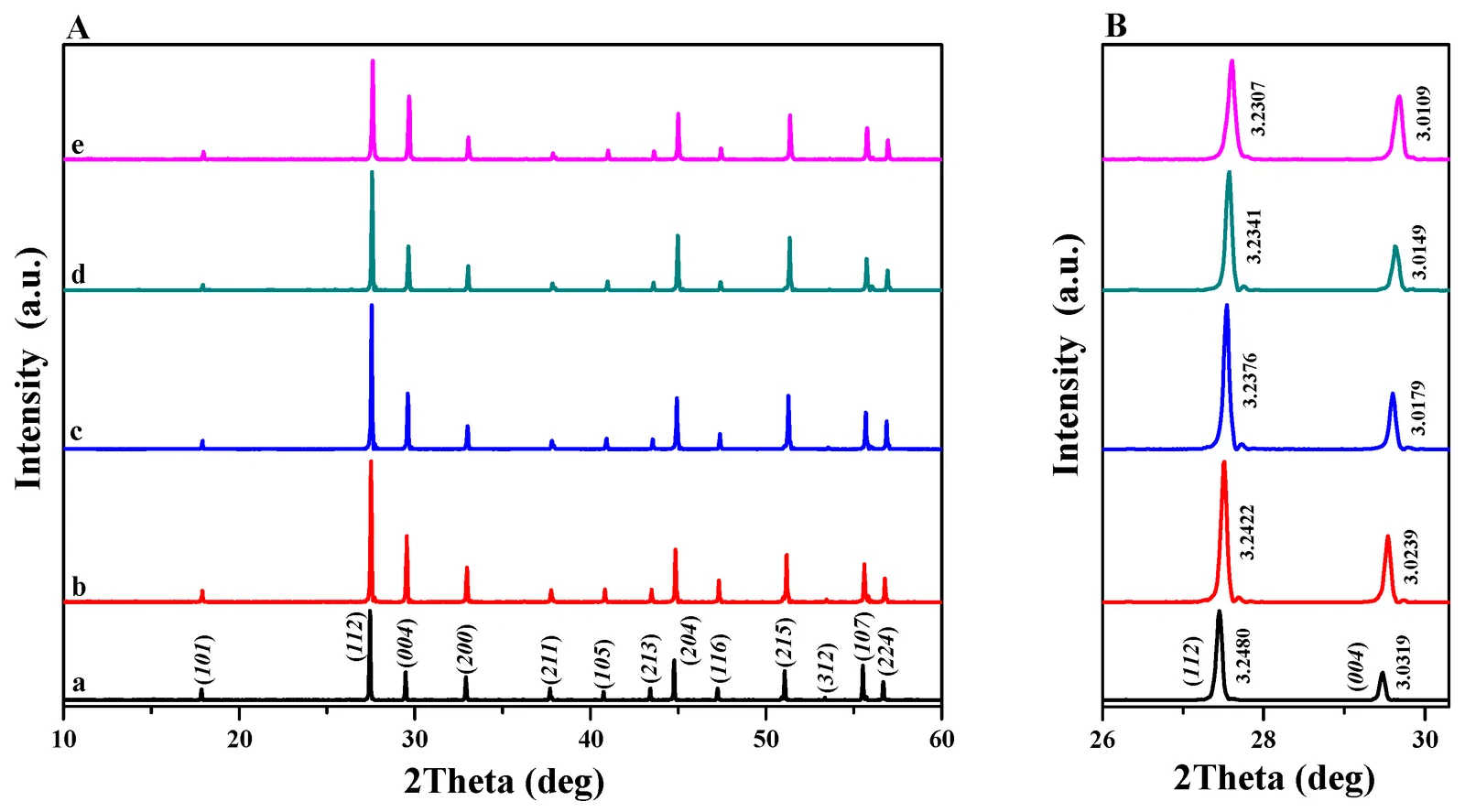
(**A**) Powder XRD patterns of PbMoO_4_ (a) and PbErMoWO samples when *x* = 0.0098 (b), *x* = 0.0283 (c), *x* = 0.0455 (d), *x* = 0.0536 (e); (**B**) powder diffraction patterns in the 2Theta range from 26 to 30.5° with (112) and (004) diffraction lines, as well as their corresponding interplanar distances.

Powder diffraction patterns of PbWO_4_ and PbErWO samples with different concentrations of Er^3+^ ions, i.e., when *x* = 0.0098 and 0.0283, are shown in Figure 2A,B. Figure 3A,B present the powder X-ray diffraction patterns of PbMoO_4_ and the PbErMoWO solid solution samples with *x* = 0.0098, 0.0283, 0.0455, and 0.0536. The XRD results of Er^3+^-doped materials showed that these patterns consisted of diffraction lines that can be assigned to a scheelite-type framework only. No impurity compounds*,* i.e.*,* initial reactants, metal oxides (PbO, Er_2_O_3_, MoO_3_, and WO_3_), and other known erbium tungstates or molybdates (Er_2_XO_6_ or Er_6_XO_12_, where X = W or Mo) were observed in PbErWO and PbErMoWO samples after their thermal treatment. All diffraction lines assigned to the scheelite-type structure shifted slightly toward higher 2Theta values with increasing Er^3+^ ion content. This can be seen in Figure 2B and Figure 3B, which show the powder diffraction patterns of the samples over a narrow range. All observed peaks were successfully indexed to a pure tetragonal scheelite-type structure (space group *I*4_1_/*a*, No. 88, PbWO_4_–JCPDs No. 01-070-7590, and PbMoO_4_–JCPDs No. 01-085-6618). The calculated lattice constants are presented in Table S1. As the Er^3+^ ion concentration increases, both lattice parameters (a and c) decrease. Thus, a contraction of the crystal lattice is observed in both solid solutions, which results from the substitution of the Pb^2+^ ions, characterized by a large ionic radius (CN = 8, i.r. = 1.29 Å), by the considerably smaller Er^3+^ ions (CN = 8, i.r. = 1.004 Å) [32]. This substitution is non-equivalent and proceeds according to the following relationship: 3 Pb^2+^ → 2 Er^3+^ + ▯. The substitution of Mo^6+^ by W^6+^ ions also occurs in the PbMoO_4_ structure.

However, the ionic radii of both ions in tetrahedral coordination are very similar (W^6+^ i.r. = 0.42 Å, Mo^6+^ i.r. = 0.41 Å), and this substitution does not significantly affect the crystal lattice of the PbErMoWO solid solution [32].

In our previous studies, we obtained analogous vacancy-type solid solutions with a scheelite-type structure, such as Ca_1-3*x*_-*_y_*Mn*_y_*▯*_x_*RE_2*x*_(MoO_4_)_1-3*x*_(WO4)_3*x*_ (RE = Pr, Nd, Eu or Gd) [35,36,37]. Similarly, in these materials, the larger Ca^2+^ ions (i.r. = 1.12 Å) were substituted with Eu^3+^ (i.r. = 1.066 Å) or Gd^3+^ (i.r. = 1.053 Å) ions, which have smaller ionic radii. In contrast to the PbErWO and PbErMoWO samples, the Ca-containing scheelite-type solid solutions which we investigated in our previous studies exhibited pronounced lattice expansion with increasing RE^3+^ concentration [35,36,37]. This was manifested by a systematic increase in the unit cell parameters or unit cell volume with increasing dopant concentration.

Table S1 also reports the experimentally determined density of the PbErWO and PbErMoWO samples, along with the respective theoretical density calculated using lattice parameters. With increasing Er^3+^ ion concentration, the density of the PbErWO solid solution samples decreases, which can be attributed to the substitution of the heavier Pb^2+^ ions by the lighter Er^3+^ ions. Additionally, the measured density is slightly lower than the theoretically calculated value, indicating that some crystallographic sites of Pb^2+^ in the PbErWO solid solution structure remain unoccupied. The density of the PbErMoWO samples increases with increasing Er^3+^ concentration. This apparent increase in density can be attributed to the competing substitution effects, namely, the replacement of Pb^2+^ by Er^3+^, which tends to decrease the density, and the substitution of the heavier W^6+^ ions for the lighter Mo^6+^ ions in the PbMoO_4_ structure, which has the opposite effect. The observed increase in density indicates that the latter effect predominates.

### 3.2. Morphology and Thermal Properties of PbErWO and PbErMoWO Solid Solutions

**Figure 4:**
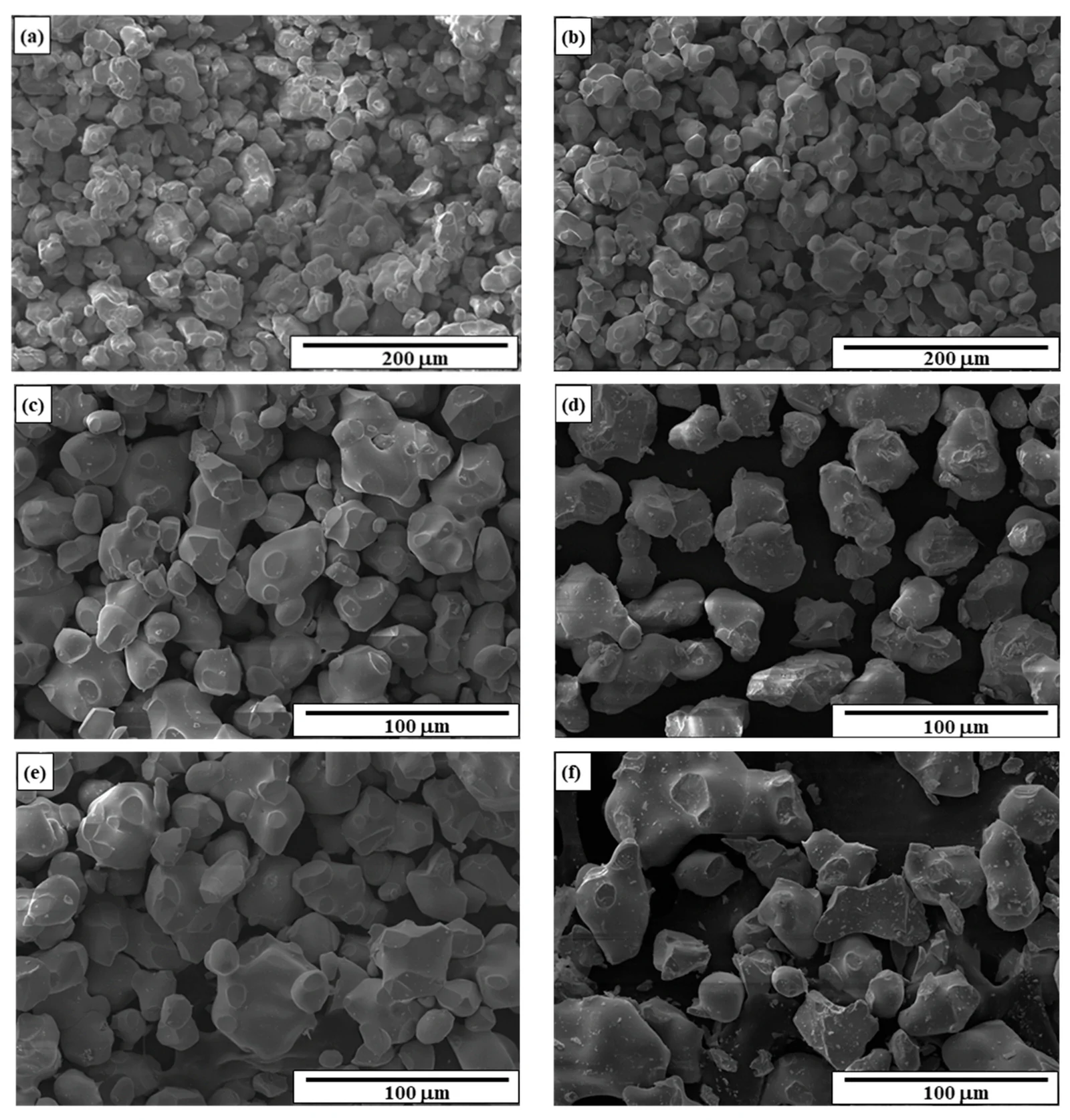
SEM images of PbWO_4_ (**a**); PbMoO_4_ (**b**); PbErWO when *x* = 0.0283 (**c**); PbErMoWO when *x* = 0.0098 (**d**); PbErMoWO when *x* = 0.0283 (**e**); PbErMoWO when *x* = 0.0536 (**f**).

SEM images of PbWO_4_, PbMoO_4_, and selected PbErWO (x = 0.0283) and PbErMoWO (*x* = 0.0098, 0.0283, and 0.0536) samples are shown in Figure 4a–f. Both matrices and all Er^3+^-doped samples are composed of grains with well-defined and sharp boundaries. This suggests that the obtained powders are well-crystallized. Samples of both matrices consist of irregularly shaped grains, predominantly oval and, to a lesser extent, spherical, with grain sizes ranging from approximately 10 to 50 μm. A fraction of the grains exhibits a tendency to agglomerate, forming large, irregularly shaped clusters with dimensions approaching 100 μm. Such large agglomerates are more pronounced in the PbWO_4_ than in the PbMoO_4_ sample.

The morphology of the Er^3+^-doped samples does not differ significantly from that observed for PbWO_4_ and PbMoO_4_. The samples predominantly exhibit large, oval-shaped grains with sizes close to 50 µm. However, in the case of the PbErMoWO solid solution sample with *x* = 0.0536, individual particles with irregular shapes and sharp edges can be observed, with sizes approaching 100 µm. This sample represents a saturated solid solution composition, and obtaining it as a single-phase material required prolonged heating, which promoted grain growth.

Energy-dispersive X-ray spectroscopy (EDS) analysis (not shown here) confirmed that the PbErWO and PbErMoWO materials contained only the elements Pb, Er, W, and O and Pb, Er, W, Mo, and O, respectively. All identified elements were uniformly distributed over the surface of the Er^3+^-doped materials, indicating a homogeneous chemical composition of each powder sample.

Knowledge of the thermal properties of new rare-earth-ion-doped tungstates and molybdate–tungstates is essential for understanding their structural stability, phase behavior, and potential applications in photoluminescent and optoelectronic devices, sensors, catalysts, or other technologies operating under elevated temperatures. The incorporation of rare-earth ions into the crystal lattice can significantly influence the thermal stability of these materials. Therefore, detailed thermal characterization is necessary to determine how the composition and dopant concentration affect their behavior upon heating.

**Figure 5:**
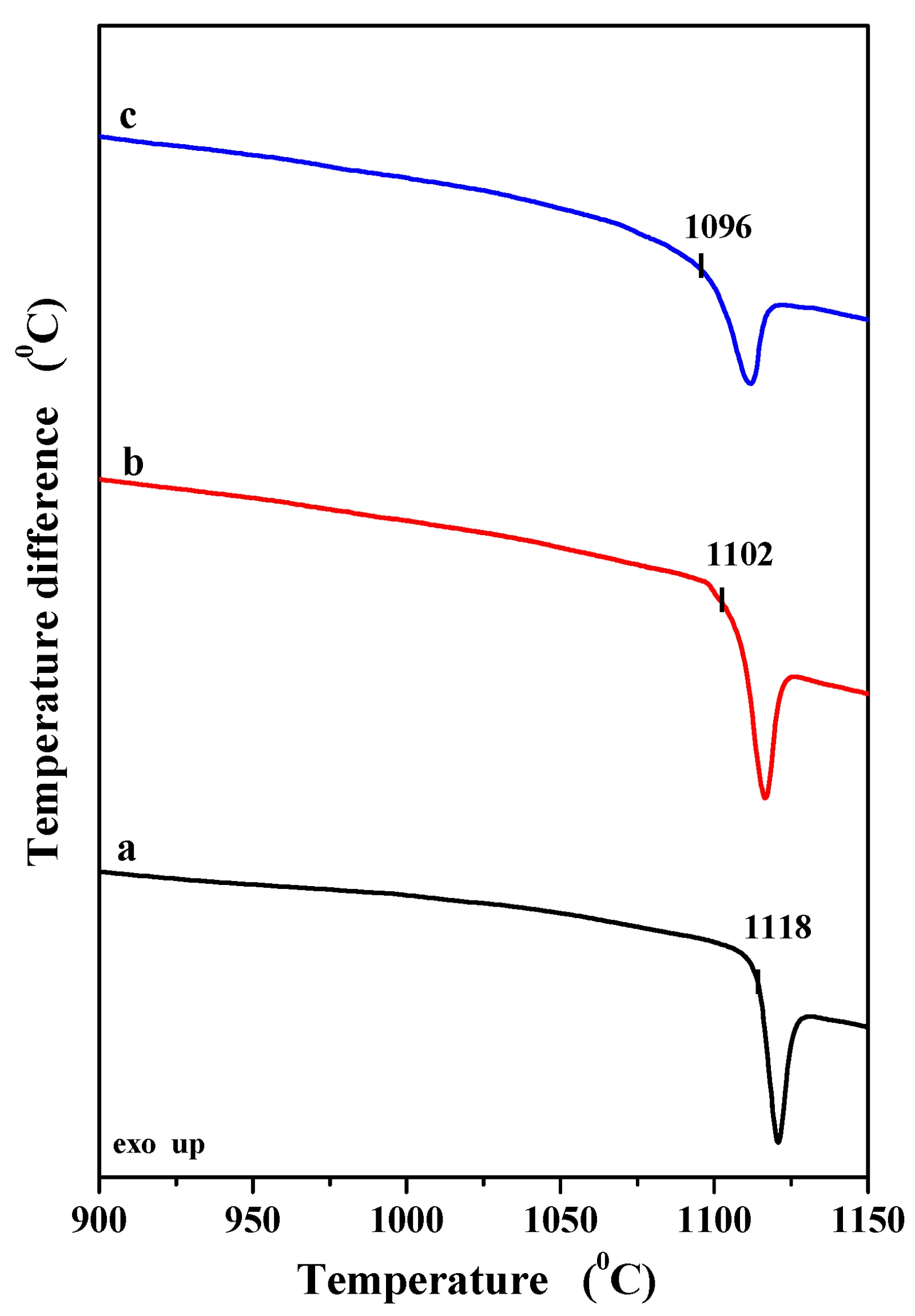
DTA curves of PbWO_4_ (a) and PbErWO samples when *x* = 0.0098 (b), *x* = 0.0283 (c).

**Figure 6:**
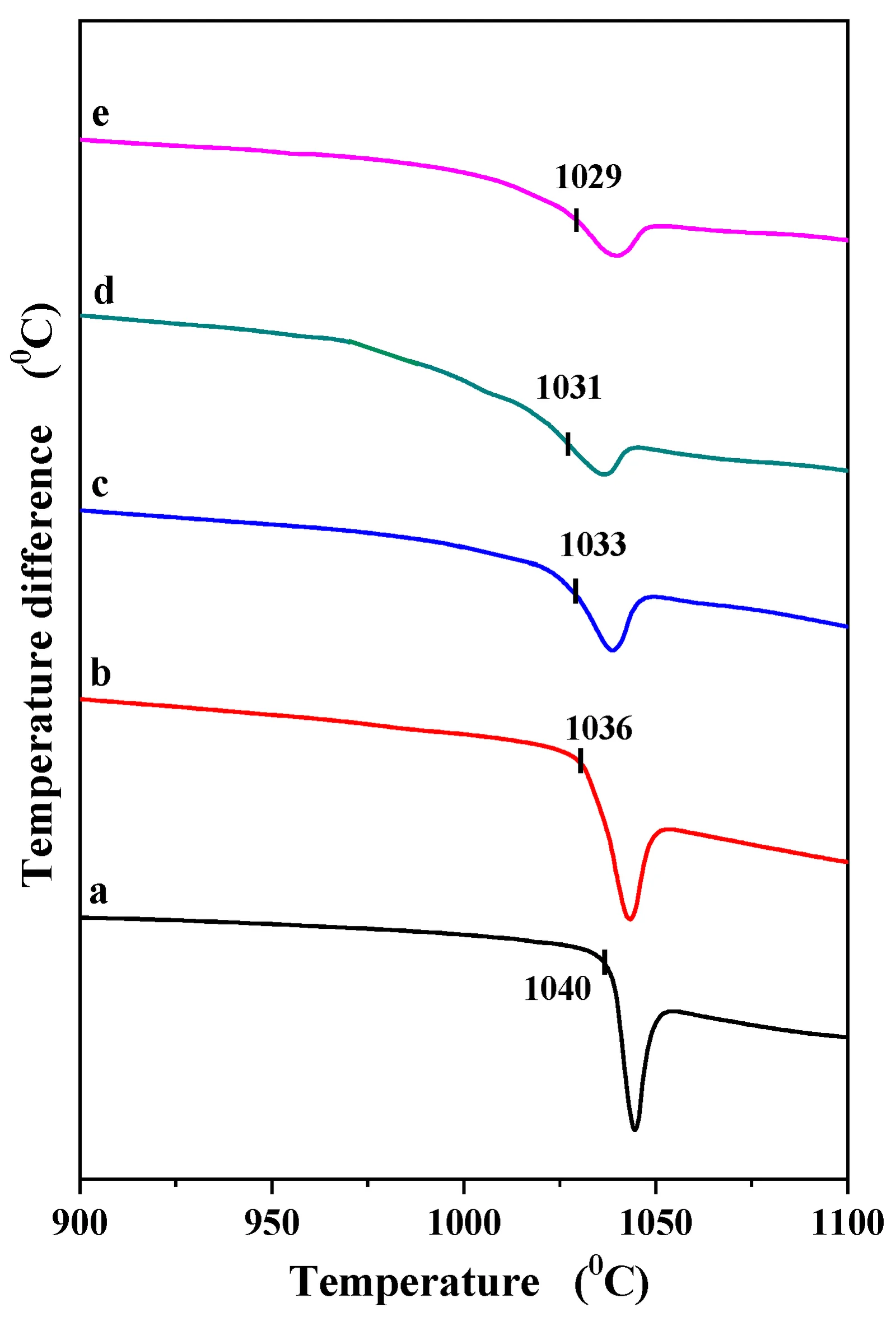
DTA curves of PbMoO_4_ (a) and PbErMoWO samples when *x* = 0.0098 (b), *x* = 0.0283 (c), *x* = 0.0455 (d), and *x* = 0.0536 (e).

In our previous studies, we demonstrated that PbWO_4_ and PbMoO_4_ melt congruently at 1118 °C and 1040 °C, respectively [29,30]. Neither compound exhibits polymorphism [29,30]. Figure 5 and Figure 6 show the DTA curves of both the matrices and the PbErWO and PbErMoWO samples. Only one endothermic effect, either symmetric or asymmetric in shape, was observed on the DTA curve for each sample of both solid solution series. Examination of the residues remaining in the measurement crucible after the DTA-DSC-TG runs confirmed that this effect corresponds to the melting of the samples. The pronounced asymmetric peaks, exhibiting a distinct inflection on the low-temperature side at a temperature slightly below the minimum of each thermal effect, may indicate the overlapping of two peaks with slightly different onset temperatures.

Such behavior was observed for the PbErWO sample with *x* = 0.0283 and for the PbErMoWO samples with *x* = 0.0283, 0.0455, and 0.0536 (Figure 5 and Figure 6). Thus, the temperatures corresponding to the overlapping endothermic effects define the solidus line (the first effect, occurring at the lower temperature) and the liquidus line (the second effect) in the phase diagrams (not shown here) of the binary PbWO_4_–Er_2_(WO_4_)_3_ and PbMoO_4_–Er_2_(WO_4_)_3_ systems. In the whole homogeneity range of both solid solutions, i.e., 0 < x ≤ 0.0283 for PbErWO and 0 < x ≤ 0.0536 for PbErMoWO, the melting point of the investigated samples decreases with increasing Er^3+^ ion concentration and is up to 20 °C lower than the melting point of the corresponding matrix.

### 3.3. Optical Properties of PbErWO and PbErMoWO Materials

The optical properties of PbWO_4_, PbMoO_4_, PbErWO, and PbErMoWO microcrystalline samples were investigated at room temperature using UV-Vis-NIR diffuse reflectance spectroscopy. This method allows the determination of very important optical parameters such as the absorption coefficient, band gap energy, Urbach energy, and refractive index. Knowledge of such parameters is important from the point of view of future applications of these materials in optoelectronics. Many divalent metal tungstates and molybdates with a scheelite-type crystal structure (e.g., MMo(W)O_4_, where M = Ca, Sr, Ba, or Cd) exhibit a characteristic absorption process associated with direct electronic transitions from the highest-energy states of the valence band to the lowest-energy states of the conduction band. Owing to the absence of impurity levels within the band gap, molybdates and tungstates are characterized by relatively high band gap energies (E_g_) [38]. Other scheelite-type compounds, i.e., lead tungstate and lead molybdate, exhibit optical absorption dominated by an indirect electronic transition [39,40]. In this mechanism, an electron is excited from the highest-energy state in the valence band to the lowest-energy state in the conduction band at a different point in the Brillouin zone. As a result, the transition requires the participation of a phonon to conserve momentum, making it a phonon-assisted process [39,40].

The lower band gap of PbMoO_4_ compared to PbWO_4_ originates from the different electronic structures of the MoO_4_^2−^ and WO_4_^2−^ tetrahedra. In PbMoO_4_, the conduction band is mainly derived from Mo 4d orbitals, whereas in PbWO_4_, it is formed predominantly by W 5d orbitals. The Mo 4d states lie at lower energies than the W 5d states, reducing the energy separation between the valence band (mainly O 2p states with some Pb 6s contribution) and the conduction band. Consequently, PbMoO_4_ exhibits a smaller band gap than PbWO_4_ [29,30,39,40]. The indirect band gaps of PbWO_4_ and PbMoO_4_ were determined in our previous studies and were found to be 3.73(2) eV and 3.17(1) eV, respectively [29,30].

**Figure 7:**
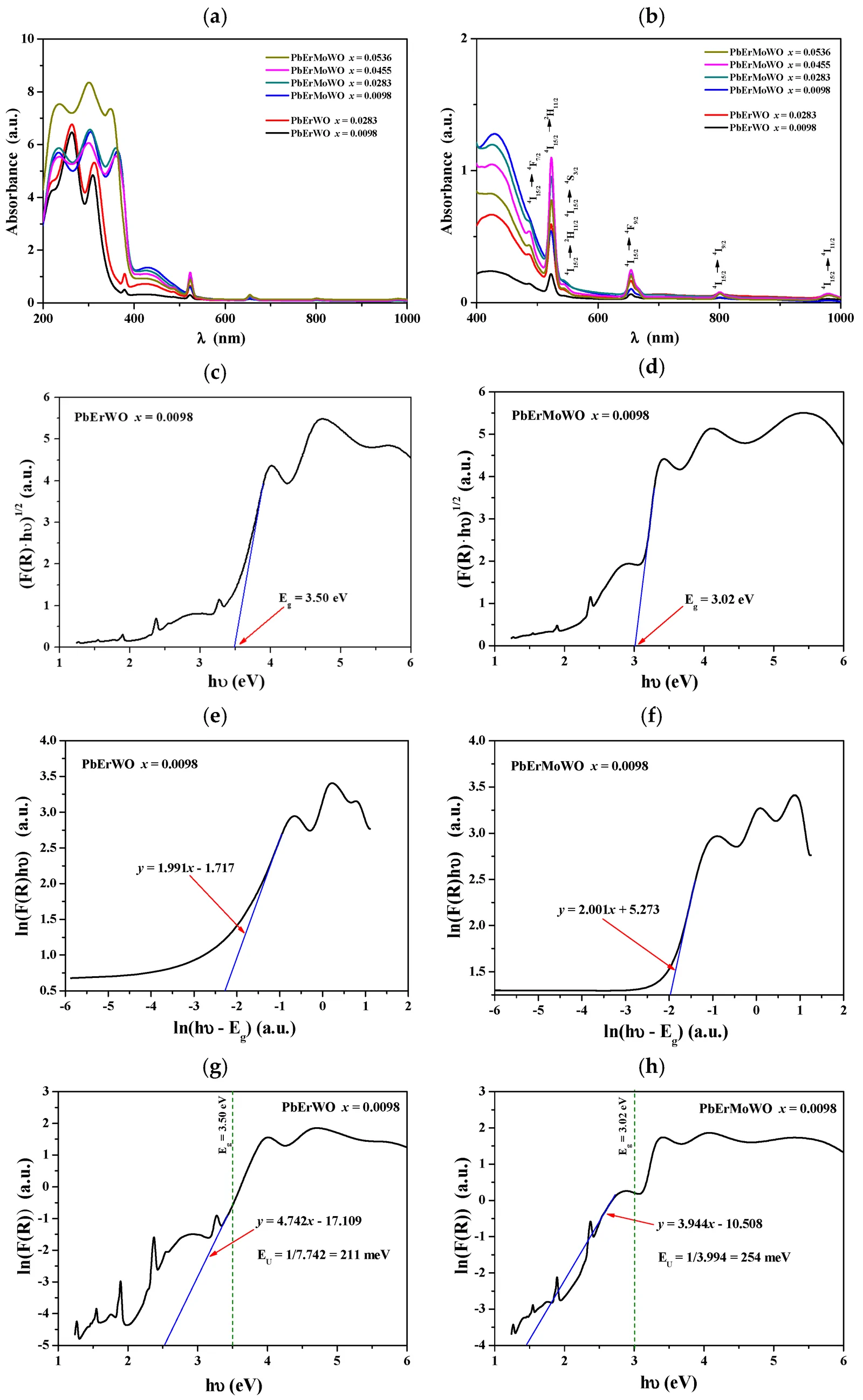
UV-Vis-NIR absorbance spectra of PbErWO and PbErMoWO samples (**a**,**b**); plots of (F(R)hν)^1/2^ versus hν of PbErWO and PbErMoWO samples when *x* = 0.0098 (**c**,**d**); plots of ln(F(R)hν) versus ln(hν-E_g_) of PbErWO and PbErMoWO samples when *x* = 0.0098 (**e**,**f**); plots of ln(F(R)) versus hν of PbErWO and PbErMoWO samples when *x* = 0.0098 (**g**,**h**).

Figure 7a,b show UV-Vis-NIR absorption spectra recorded at ambient temperature for PbErWO and PbErMoWO samples. All obtained materials show energy absorption ability in the ultraviolet, visible, and NIR ranges. The very intense and broad absorption bands with the spectral range of 200–370 nm can be assigned to O^2−^ → W^6+^ and O^2−^ → Mo^6+^ charge transfer bands in WO_4_ and MoO_4_, respectively [29,30,35,36,37]. Also, intense but narrow bands observed at higher wavelengths (visible and NIR region) can be attributed to the transitions from the ground state ^4^I_15/2_ of the Er^3+^ ion to the excited states (Figure 7b) [41,42,43,44]. In this work, we focused exclusively on the analysis of absorption spectra to determine the optical band gap, Urbach energy, and refractive index.

The indirect band gap energy was determined based on UV–Vis–NIR diffuse reflectance spectroscopy (DRS) measurements. The recorded spectra were expressed as F(R) = f(λ), where R represents the diffuse reflectance of the investigated material and λ denotes the wavelength. They covered the absorption edge within the investigated spectral range. Subsequently, the Kubelka–Munk function was employed to convert the data obtained from UV–VIS–NIR DRS measurements into a parameter that can be regarded as an approximation of the absorption coefficient. The Kubelka–Munk approach is particularly applicable to powdered and optically opaque samples, for which the conventional determination of the absorption coefficient from transmittance measurements is challenging or, in many cases, not feasible. This function is defined as follows [45]:

(3) $$F\left(R\right)=\frac{{\left(1-R\right)}^{2}}{2R}$$

The optical band gap was determined using the Tauc relation [46,47].

(4) $$F(R)h\nu =A{\left(h\nu -E_{g}\right)}^{n}$$

where F(R) is the Kubelka–Munk function of the material under study, h is Planck’s constant, ν is the light frequency, A is a proportionality coefficient characteristic of a material, and n is a constant related to the nature of the electronic transition [46,47]. For solid materials with an indirect band gap, n = 2 [46,47]. The values of E_g_ were determined from the plot of (F(R)hν)^1/2^ as a function of hν by extrapolating the linear portion of this relation to the photon energy axis as shown in Figure 7c,d for the PbErWO and PbErMoWO samples (*x* = 0.0098), respectively. The Eg values determined for all samples are summarized in Table S2, along with equations of the tangent lines to (F(R)hν)^1/2^ vs. hν plots with linear fitting range. The indirect band gap energies of the samples for both solid solutions are lower than those of the corresponding pure matrices and change nonlinearly with increasing Er^3+^ ion concentration. The systematic change in the optical band gap with Er^3+^ doping is connected with structural disorder and on-site fluctuations, which arise due to the substitution of Er^3+^ for Pb^2+^ and W^6+^ for Mo^6+^ in the framework of PbMoO_4_, as well as the creation of vacancies according to the following substitution: 3 Pb^2+^ → 2 Er^3+^ + ▯. Additionally, there is a significant difference in electronegativity between Pb (2.33) and Er (1.24), as well as Mo (2.16) and W (2.36). The differences in the electronegativity values shift the conduction band toward the valence band and lead to a decrease in the band gap with doping.

To confirm the type of electronic transition in the studied materials, plots of ln(F(R)hν) versus ln(hν − E_g_) were constructed, and the value of n was determined from the slope of these curves. The corresponding plots for the PbErWO and PbErMoWO samples with *x* = 0.0098 are shown in Figure 7e,f, respectively. The obtained values of n, 1.991 and 2.001, respectively, are very close to 2, clearly indicating that the Er^3+^-doped scheelite-type tungstates and molybdate–tungstates exhibit an indirect band gap.

The Urbach energy (E_U_) is a parameter that characterizes the exponential absorption tail, known as the Urbach tail, extending below the fundamental optical band gap of a semiconductor or insulating material. It is a measure of the degree of structural and electronic disorder in a material and reflects the distribution of localized electronic states near the band edges. The optical absorption coefficient in the Urbach region follows the empirical relation [48,49,50]:

(5) FR=F0R exph−E0EU

where F(R) is the Kubelka–Munk function, hν is the photon energy, E_U_ is the Urbach energy, and F_0_(R) and E_0_ are material-dependent constants.

A low Urbach energy (10–50 meV) for high-quality crystalline semiconductors indicates a sharp absorption edge and a low density of defect-induced localized states, reflecting good crystallinity and minimal structural disorder. In contrast, a high Urbach energy (in the order of 200–300 meV) is generally considered evidence of significant disorder within the material. The large values suggest a high concentration of structural defects and imperfections, substantial lattice disorder or partial amorphization, pronounced compositional fluctuations or chemical inhomogeneity, a large density of localized electronic states extending into the band gap, and increased electron–phonon interactions and band-edge fluctuations [48,49,50]. Consequently, materials exhibiting an Urbach energy above 200 meV possess a broad distribution of tail states that facilitate sub-band gap optical absorption and often act as trapping or recombination centers for charge carriers [48,49,50]. The Urbach energy was determined from the exponential dependence of the absorption coefficient on photon energy near the absorption edge. It was obtained by fitting the linear region of a plot of ln(α) as a function of photon energy, as shown in Figure 7g,h for PbErWO and PbErMoWO samples with *x* = 0.0098. The calculated Urbach energies for the studied samples are summarized in Table S2, along with equations of the tangent lines to ln(F(R)) vs. hν plots with Urbach energy fitting range. As shown, the evolution of the optical band gap exhibits an inverse relationship with the degree of structural disorder. Consequently, a reduction in E_g_ is accompanied by a broadening of the Urbach tail. The E_U_ values obtained for the PbErWO and PbErMoWO powders are significantly higher than those determined for the undoped PbWO_4_ and PbMoO_4_ samples. Moreover, they reach values in the range of 200–300 meV, clearly indicating a high degree of structural disorder and a higher concentration of defect states in the solid solutions compared with the pure matrices.

The determination of the refractive index (n) is an important aspect of the optical characterization of powdered materials. The refractive index is closely related to the chemical composition of materials, their density, electronic polarizability, and structural properties, making it a valuable parameter for material identification and characterization. In the case of powders, refractive index measurements are particularly challenging because the optical response is influenced by the heterogeneous nature of these samples. Particle size, morphology, porosity, and packing density may significantly affect light scattering and the measured optical properties. Therefore, appropriate measurement methods and careful interpretation of the results are essential. Indirect methods for determining the refractive index involve measuring other physical quantities, from which the refractive index can subsequently be estimated. Several empirical models have been developed that allow the refractive index to be estimated based on the known value of the band gap energy (E_g_). These include, among others, the following models:

1. Moss model for 0 < E_g_ < 3.67 eV. This is an empirical model that provides an estimate of the refractive index. It is primarily reliable for semiconductors, although the relationship is also sometimes used as an approximate estimate for insulators, oxides, and glasses. It may produce significant errors, particularly for materials with an atypical band structure, a very small energy gap, or optical properties that deviate from those of typical semiconductors [51].

(6) $$n={\left(\frac{95}{E_{g}}\right)}^{\frac{1}{4}}$$

2. Harvé–Vandamme model for 2.0 < E_g_ < 4.0 eV. The model is an empirical relation used to estimate the refractive index of semiconductors, excluding IV–VI semiconductors such as PbS, PbSe, PbTe, SnSe, and SnTe [52].

(7) $$n=\sqrt{1+{\left(\frac{13.6}{E_{g}+3.4}\right)}^{2}}$$

3. Reddy–Ahammed model for 0.365 ≤ E_g_ < 6.20 eV. The model is an empirical relation for estimating the refractive index of semiconductors; however, it does not accurately predict the refractive indices of certain materials, including PbS, PbSe, PbTe, and InSb [53].

(8) $$n={\left(\frac{154}{E_{g}-0.365}\right)}^{\frac{1}{4}}$$

4. Reddy–Anjaneyulu model without specifying a range of E_g_ values. The model is an empirical relationship used to estimate the refractive index of materials, primarily semiconductors. It may exhibit significant discrepancies with experimental data in certain band gap energy ranges. It should not be regarded as a universal model applicable to all materials [54].

(9) $$n=\ln\left(\frac{36.3}{E_{g}}\right)$$

5. Kumar–Singh model for 0.1 eV ≤ E_g_ ≤ 8.5 eV. The authors validated the model for 34 different materials (semiconductors, insulators, oxides, and halides) with band gaps ranging from 0.1 to 8.5 eV, achieving agreement with experimental data at an R value of 0.9133 [55].

(10) $$n=\frac{3.3668}{E_{g}^{0.32234}}$$

6. Dimitrov–Sakka model without specifying a range of E_g_ values. It is an empirical relationship used to estimate the refractive index, primarily of simple oxides and oxide glasses (silicates, borates, phosphates, germanates, and tellurates). The accuracy of the model depends on the chemical composition and structure of the material under investigation, as well as the polarizability of the ions it contains [56].

(11) $$n=\sqrt{\frac{3-2\sqrt{\frac{E_{g}}{20}}}{\sqrt{\frac{E_{g}}{20}}}}$$

7. Tripathy model for 2.0 eV ≤ E_g_ ≤ 6.0 eV. The model can be used to empirically estimate the refractive index of binary and ternary semiconductors, as well as alkali halides [57].

(12) $$n=1.73\left(1+1.9017\cdot e^{-0.539E_{g}}\right)$$

**Figure 8:**
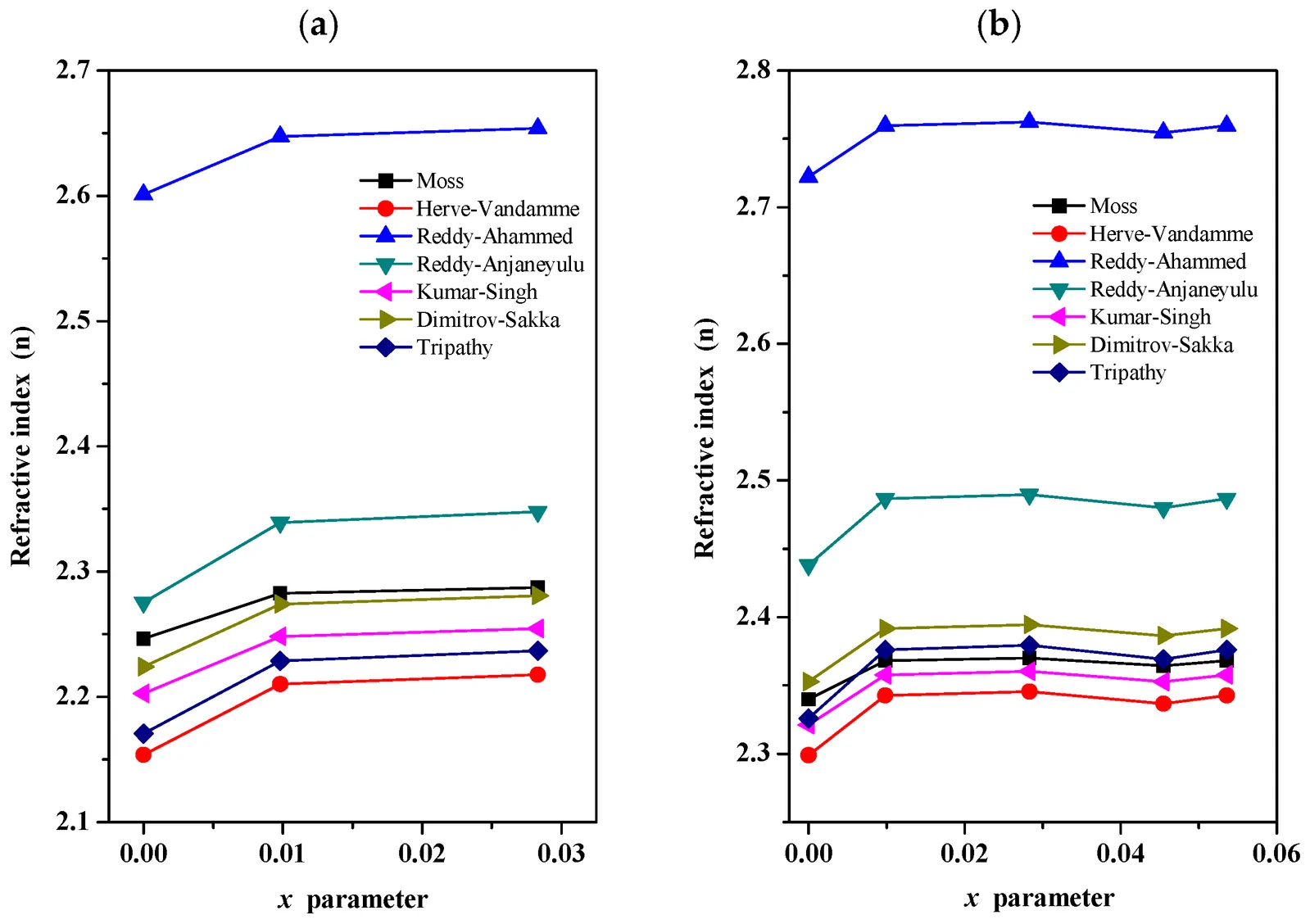
Refractive index (n) estimated using seven models as a function of Er^3+^ concentration of PbErWO (**a**) and PbErMoWO (**b**) samples.

Figure 8a,b present the estimated refractive index as a function of the Er^3+^ ion concentration for the PbErWO and PbErMoWO samples, respectively. The corresponding refractive index values are summarized in Table S3. Seven well-known empirical models were used to estimate the refractive index (n), including the Moss model, even though the host matrix of the PbErWO solid solution, i.e., PbWO_4_, does not satisfy the E_g_ criterion. For this sample, the indirect band gap is 3.73 eV, which lies outside the range of applicability specified by the author of the model. As evident from Figure 8a,b, the refractive index exhibits only a weak dependence on the *x* parameter. For the six models considered, the estimated values of (n) fall within the ranges of 2.15–2.35 and 2.30–2.50 for the PbErWO and PbErMoWO samples, respectively. A distinct deviation is observed for the Reddy–Ahammed model, which yields refractive index values approximately 0.30 higher than those obtained from the remaining models for both series of solid solutions. The relatively high refractive indices (n > 2) and wide indirect band gaps (E_g_ > 3 eV) of the PbErWO and PbErMoWO micropowders suggest that these materials may be of interest for potential applications in optoelectronic and optical-sensing technologies, such as optical interference filters and optical sensors. However, further investigation of their optical losses, dispersion, and other application-specific properties is required to assess their suitability for particular devices.

## 4. Conclusions

Er^3+^-doped PbWO_4_- and PbMoO_4_-based solid solutions were successfully obtained via a conventional high-temperature solid-state route. PXRD analysis confirmed that Er^3+^ incorporation does not lead to the formation of secondary crystalline phases and that all investigated compositions retain the tetragonal scheelite-type structure with the *I*4_1_/*a* space group. The maximum Er^3+^ contents incorporated into the studied matrices were *x* = 0.0283 for PbErWO and *x* = 0.0536 for PbErMoWO.

A gradual decrease in the unit cell parameters with increasing Er^3+^ content was observed for both series. This behavior is consistent with the replacement of Pb^2+^ by the smaller Er^3+^ ions and the concomitant formation of Pb^2+^-site vacancies required for charge compensation. In the PbErMoWO solid solution, the additional Mo^6+^/W^6+^ substitution has a comparatively small influence on the lattice dimensions because of the close ionic radii of these two tetrahedrally coordinated cations.

Microscopic observations revealed well-crystallized powders consisting mainly of irregular and oval-shaped grains. Er^3+^ incorporation did not cause pronounced changes in the overall morphology, while the highest Er^3+^ content in the PbErMoWO series was associated with noticeable grain growth. EDS analysis confirmed the expected elemental composition and a homogeneous distribution of the constituent elements throughout the examined samples.

Thermal analysis showed that the investigated materials remain structurally stable up to their melting point and do not undergo polymorphic transformation. Increasing Er^3+^ concentration results in a gradual lowering of the melting point.

The optical measurements demonstrated that all Er^3+^-containing materials exhibit indirect optical transitions. Er^3+^ incorporation leads to a progressive reduction in the optical band gap energy and a simultaneous increase in the Urbach energy. These changes indicate increasing structural and electronic disorder within the solid solutions, associated with aliovalent substitution, cation-vacancy formation, and, in the PbErMoWO system, partial replacement of Mo^6+^ by W^6+^. The estimated refractive indices show only a weak dependence on Er^3+^ concentration and remain relatively high for both material series.

Overall, the obtained results demonstrate that Er^3+^ incorporation provides an effective means of modifying the structural, thermal, and optical characteristics of PbWO_4_- and PbMoO_4_-based scheelite-type materials. The established relationships between composition, crystal structure, thermal behavior, and optical parameters provide a basis for further investigations of these solid solutions in the context of optical and optoelectronic applications.

## Data Availability

The original contributions presented in this study are included in the article/Supplementary Material. Further inquiries can be directed to the corresponding author.

## Supplementary Materials

The following supporting information can be downloaded at: https://www.mdpi.com/article/10.3390/ma19214661/s1, Table S1: Composition of initial Er2(WO4)3/PbWO4 and Er2(WO4)3/PbMoO4 mixtures, formula of PbErWO and PbErMoWO solid solutions, calculated a and c lattice parameters, c/a ratio, and determined (dexp) and calculated (dcal) density. Table S2: Chemical formulas of PbErWO and PbErMoWO solid solutions, indirect band gap energy (Eg), Urbach energy (EU), equations of the tangent lines to (F(R)hν)1/2 vs. hν plots with linear fitting range, and equations of the tangent lines to ln(F(R)) vs. hν plots with Urbach energy fitting range. Table S3: Chemical formulas of PbErWO and PbErMoWO solid solutions and estimated refractive index (n) using the Moss, Hervé–Vandamme, Reddy–Ahammed, Reddy–Anjaneyulu, Kumar–Singh, Dimitrov–Sakka, and Tripathy models.
