# Supplementary material for: Structural, Thermal, and Optical Properties of Er3+-Doped Lead Tungstates and Lead Molybdate–Tungstates
Source: Materials (Basel). 2026 Nov 2;19(21):4661. doi: 10.3390/ma19214661 (PMC13765082; doi:10.3390/ma19214661)
Supplement: Supplementary file 1 [file materials-19-04661-s001.zip › materials-4619937-supplementary.pdf]

Table S1. Composition of initial $Er_2(WO_4)_3/PbWO_4$ and $Er_2(WO_4)_3/PbMoO_4$ mixtures, formula of PbErWO and PbErMoWO solid solutions, calculated *a* and *c* lattice parameters, *c*/*a* ratio, and determined (d_{exp}) and calculated (d_{cal}) density.

$Er_2(WO_4)_3$ content [mol%]	$PbWO_4$ or $PbMoO_4$ content [mol%]	Formula of PbErWO or PbErMoWO solid solution, values of *x* parameter		Lattice parameters			d_{exp} [g cm^{-3}]	d_{cal} [g cm^{-3}]
				a [Å]	*c* [Å]	*c*/*a*		
0	100.00	$x = 0$	$PbWO_4$	5.46659(8)	12.0572(11)	2.2056	8.41(1)	8.39
1.00	99.00	$x = 0.0098$	$Pb_{0.9706}□_{0.0098}Er_{0.0196}WO_4$	5.45840(6)	12.0389(13)	2.2056	8.30(2)	8.37
3.00	97.00	$x = 0.0283$	$Pb_{0.9151}□_{0.0283}Er_{0.0566}WO_4$	5.44861(6)	12.0165(9)	2.2054	8.27(2)	8.32
0	100.00	$x = 0$	$PbMoO_4$	5.44105(5)	12.1196(13)	2.2274	6.80(2)	6.80
1.00	99.00	$x = 0.0098$	$Pb_{0.9706}□_{0.0098}Er_{0.0196}(MoO_4)_{0.9706}(WO_4)_{0.0294}$	5.43441(8)	12.0956(11)	2.2257	6.81(1)	6.82
3.00	97.00	$x = 0.0283$	$Pb_{0.9151}□_{0.0283}Er_{0.0566}(MoO_4)_{0.9151}(WO_4)_{0.0849}$	5.42971(7)	12.0749(10)	2.2238	6.84(2)	6.84
5.00	95.00	$x = 0.0455$	$Pb_{0.8635}□_{0.0455}Er_{0.0910}(MoO_4)_{0.8635}(WO_4)_{0.1365}$	5.42478(5)	12.0555(11)	2.2223	6.85(1)	6.85
6.00	94.00	$x = 0.0536$	$Pb_{0.8392}□_{0.0536}Er_{0.1072}(MoO_4)_{0.8392}(WO_4)_{0.1608}$	5.42450(9)	12.0528(13)	2.2219	6.86(2)	6.85

Table S2. Chemical formulas of PbErWO and PbErMoWO solid solutions, indirect band gap energy (E_g), Urbach energy (E_U), equations of the tangent lines to $(F(R)h\nu)^{1/2}$ vs. hν plots with linear fitting range, and equations of the tangent lines to ln(F(R)) vs. hν plots with Urbach energy fitting range.

Formula of PbErWO or PbErMoWO solid solution	Values of *x* parameter	E_g [eV]	Equations of the tangent lines to $(F(R)h\nu)^{1/2}$ vs. hν plots<br>Linear fitting range	E_U [meV]	Equations of the tangent lines to ln(F(R)) vs. hν plots<br>Urbach energy fitting range
x = 0	$PbWO_4$	3.73(2)	y = 17.983 x – 67.339<br>3.83 – 3.97 eV	188(5)	y = 5.319 x – 17.000<br>3.56 – 3.67 eV
x = 0.0098	$Pb_{0.9706}\square_{0.0098}Er_{0.0196}WO_4$	3.50(1)	y = 9.456 x – 32.973<br>3.81 – 3.89 eV	211(9)	y = 4.742 x – 17.109<br>3.35 – 3.43 eV
x = 0.0283	$Pb_{0.9151}\square_{0.0283}Er_{0.0566}WO_4$	3.47(2)	y = 10.041 x – 34.143<br>3.70 – 3.78 eV	257(7)	y = 3.891 x – 13.334<br>3.33 – 3.45 eV
x = 0	$PbMoO_4$	3.17(1)	y = 4.079 x – 10.707<br>3.35 – 3.48 eV	223(6)	y = 4.484 x – 12.638<br>2.21 – 2.50 eV
x = 0.0098	$Pb_{0.5710}\square_{0.1430}Er_{0.2860}(MoO_4)_{0.5710}(WO_4)_{0.4290}$	3.02(2)	y = 13.977 x – 42.278<br>3.22 – 3.29 eV	254(6)	y = 3.944 x – 10.508<br>2.52 – 2.70 eV
x = 0.0283	$Pb_{0.5710}\square_{0.1430}Er_{0.2860}(MoO_4)_{0.5710}(WO_4)_{0.4290}$	3.01(2)	y = 12.543 x – 37.587<br>3.20 – 3.29 eV	263(5)	y = 3.802 x – 10.344<br>2.60 – 2.73 eV
x = 0.0455	$Pb_{0.5710}\square_{0.1430}Er_{0.2860}(MoO_4)_{0.5710}(WO_4)_{0.4290}$	3.04(1)	y = 15.222 x – 46.254<br>3.19 – 3.27	275(6)	y = 3.636 x – 10.093<br>2.60 – 2.73 eV
x = 0.0536	$Pb_{0.5710}\square_{0.1430}Er_{0.2860}(MoO_4)_{0.5710}(WO_4)_{0.4290}$	3.02(1)	y = 11.133 x – 33.578<br>3.21 – 3.40	289(7)	y = 3.460 x – 9.804<br>2.60 – 2.73 eV

Table S3. Chemical formulas of PbErWO and PbErMoWO solid solutions and estimated refractive index (n) using the Moss, Hervé–Vandamme, Reddy–Ahammed, Reddy–Anjaneyulu, Kumar–Singh, Dimitrov–Sakka, and Tripathy models.

Formula of PbErWO or PbErMoWO solid solution values of x parameter		Refractive index						
		Moss	Hervé–Vandamme	Reddy–Ahammed	Reddy–Anjaneyulu	Kumar–Singh	Dimitrov–Sakka	Tripathy
$x = 0$	$PbWO_4$	2.24465	2.1537	2.6010	2.2754	2.2026	2.2241	2.1706
$x = 0.0098$	$Pb_{0.9706}\square_{0.0098}Er_{0.0196}WO_4$	2.2825	2.2102	2.6474	2.3391	2.2482	2.2741	2.2288
$x = 0.0283$	$Pb_{0.9151}\square_{0.0283}Er_{0.0566}WO_4$	2.2874	2.2179	2.6538	2.3477	2.2545	2.2809	2.2369
$x = 0$	$PbMoO_4$	2.3397	2.2989	2.7221	2.4381	2.3212	2.3527	2.3259
$x = 0.0098$	$Pb_{0.5710}\square_{0.1430}Er_{0.2860}(MoO_4)_{0.5710}(WO_4)_{0.4290}$	2.3683	2.3426	2.7597	2.4866	2.3577	2.3917	2.3760
$x = 0.0283$	$Pb_{0.5710}\square_{0.1430}Er_{0.2860}(MoO_4)_{0.5710}(WO_4)_{0.4290}$	2.3702	2.3455	2.7623	2.4899	2.3602	2.3944	2.3795
$x = 0.0455$	$Pb_{0.5710}\square_{0.1430}Er_{0.2860}(MoO_4)_{0.5710}(WO_4)_{0.4290}$	2.3644	2.3366	2.7545	2.4800	2.3527	2.3864	2.3691
$x = 0.0536$	$Pb_{0.5710}\square_{0.1430}Er_{0.2860}(MoO_4)_{0.5710}(WO_4)_{0.4290}$	2.3683	2.3426	2.7597	2.4866	2.3577	2.3917	2.3760
